# Effects of Pb(II) and Cr(VI) Stress on Phosphate-Solubilizing Bacteria (*Bacillus* sp. Strain MRP-3): Oxidative Stress and Bioaccumulation Potential

**DOI:** 10.3390/ijerph16122172

**Published:** 2019-06-19

**Authors:** Wen Shao, Min Li, Zedong Teng, Bin Qiu, Yaoqiang Huo, Keyao Zhang

**Affiliations:** College of Environmental Science and Engineering, Beijing Forestry University, Beijing 100083, China; shaow@bjfu.edu.cn (W.S.); zedong_teng@126.com (Z.T.); qiubin2015@bjfu.edu.cn (B.Q.); huoyaoqiang@bjfu.edu.cn (Y.H.); jdxwl_sw@126.com (K.Z.)

**Keywords:** phosphate-solubilizing bacteria, heavy metal tolerance, reactive oxygen species (ROS), antioxidative enzymes, bioaccumulation

## Abstract

The aim of this work was to ascertain the effects of Pb(II) and Cr(VI) on bacterial growth, generation of reactive oxygen species (ROS), activities of superoxide dismutase (SOD), and catalase (CAT), as well as the localization of bioaccumulated heavy metals in a phosphate-solubilizing bacterium. The results showed that the ROS increased from 1.4-fold to 1.8-fold of control under Pb(II) stress and decreased from 1.6-fold to 1.1-fold of control under Cr(VI) stress corresponding to metal concentrations (0.5–5 mmol·L^−1^). The SOD activities were ROS dependent; however, the CAT activities increased under both Pb(II) and Cr(VI) stress, from 11.4 to 21.8 U·mg^−1^ and 11.4 to 32.9 U·mg^−1^, respectively. Intra/extracellular accumulation were investigated by scanning transmission electron microscopy with energy dispersive X-ray spectroscopy (STEM-EDS) and it was calculated that extracellular accumulated Pb accounted for 61.7–95.9% of the total accumulation, while extracellular accumulated Cr only accounted for up to 3.6% of the total accumulation. Attenuated total reflection/Fourier-transform infrared spectroscopy (ATR-FTIR) analysis confirmed that the functional groups involved in those extracellular accumulation were not located in the loosely bound extracellular polysaccharides substances.

## 1. Introduction

Phosphate-solubilizing bacteria (PSB) was brought to the attention of the public because of its capacity of increasing P utilization in the context of low phosphorus utilization efficiency [1,2,3]. Additionally, PSB with high tolerance to heavy metals can diversely increase P utilization, promote plant growth, protect rhizosphere, as well as mobilize heavy metals to make plant uptake easier [4,5,6]. However, most researches of PSB were focused on isolating and assessing its ability to absorb heavy metals, with barely any study on the various effects caused by different heavy metals and bacteria-heavy metal interactions, which impede the clarification of PSB’s tolerance mechanisms against different heavy metals. Thus, one PSB strain with high heavy metal tolerance was isolated and incubated with two typical heavy metals (Pb and Cr) to conduct the research. 

Lead (Pb), which is an extremely toxic heavy metal with its primarily present as Pb(II), disturbs various organism physiological processes and does not play any biological functions [7]. Under the influence of lead, the level of the reactive oxygen species (ROS) increases and breaks the balance between the production of free radicals and the generation of antioxidants, which was thought to be a significant mechanism of lead toxicity [7,8]. Chromium (Cr) exhibits crucial biotoxicity and no biological roles either. It shows high solubility and bioavailability in the environment when being the form of Cr(VI). Cr(VI) is a strong oxidizing agent and mostly formed as CrO_4_^2−^, which can easily penetrate cell membrane through channels for isoelectric and isostructural anions, such as SO_4_^2−^ and HPO_4_^2−^ [7,9]. After Cr(VI) ions penetrate into cell, the intracellular reduction of Cr(VI) starts to operate as a detoxification mechanism. However, biological reductants reduce Cr(VI) to Cr(V) and finally to Cr(III) by utilizing a single electron donated by nicotinamide adenine dinucleotide (NADH), thus, releasing ROS [9,10]. 

The amount of ROS is relatively low under normal physiological conditions, while large amounts of ROS will be produced under heavy metal conditions and cause oxidative stress inside the cell, which is well known as a significant mechanism of cytotoxicity [11,12,13]. Excess ROS (singlet oxygen, superoxide anion, hydrogen peroxide, and hydroxyl radical) can oxidize multiple types of biomacromolecules, including unsaturated fatty acid, protein, and pigment, thus giving rise to membrane damage, enzyme inactivation, and DNA damage, which is thought to be a main approach to cell death [14,15,16]. The induction of ROS via heavy metals has been extensively reported—Hg elevated ROS formation in *Coccomyxa* [17], Pb induced oxidative stress and cytotoxicity in zebrafish [18]. To prevent damage from ROS, organisms have evolved multiple detoxification mechanisms, including the synthesis various antioxidant enzymes by which organisms responds to oxidative stress. Changes of antioxidant enzymes activity such as superoxide dismutase (SOD) and catalase (CAT) are the most important characteristics of an antioxidant system—SOD can catalyze O_2_^−^ to O_2_, H_2_O_2_ rapidly, then H_2_O_2_ is decomposed by CAT, which protects biomolecules from ROS-mediated damage [19].

Besides antioxidant enzymes, bioaccumulation was also regarded as a main approach to tolerating heavy metals. Bioaccumulation includes all processes responsible for the uptake of available metal ions by living cells. It includes biosorption, together with intracellular accumulation and precipitation mechanisms [20]. The adsorption is mainly by electrostatic attraction to opposite charged functional groups on the surface of extracellular polysaccharides substances (EPS) and cell walls—this process occurs rapidly [8,21]. Additionally, EPS was thought to be a key role in adsorption. EPS belong to the heterogeneous mixture which results from bacterial secretion, shedding of cell surface materials, cell lysis materials, and adsorption of organic matters from the environment. Bacteria have a double-layered EPS structure—loosely bound EPS and tightly bound EPS. Loosely bound EPS can be separated by centrifugation and has more important role in bacterial properties [22,23]. Thus, the investigation of loosely bound EPS was performed in this study. The intracellular accumulation occurs due to the transport systems of cell, which requires cell metabolic energy and is a much slower process. Precipitation is that interaction between metals and metabolites in the surrounding, such as precipitation of Pb(II) by phosphate and precipitation of Cr(VI) by sulphide. 

In the present study, a phosphate-solubilizing bacterium *Bacillus* sp. strain MRP-3 with high ability of Pb(II) and Cr(VI) tolerance was isolated. The impacts of Pb(II) and Cr(VI) on this strain were compared by investigating and characterizing (a) ROS generation, (b) antioxidant enzymes activities, (c) localization of bioaccumulated heavy metals, and (d) surface functional groups. It is expected to elucidate the different effects caused by Pb(II) and Cr(VI) stress on isolated strain, which might be useful in providing basic guidance for further research in bacterial resistance mechanism to heavy metals and regarding *Bacillus* sp. strain MRP-3 as a promising candidate for soil bioremediation. 

## 2. Materials & Methods

### 2.1. Isolation and Identification of Heavy Metal Resistant PSB Strain

The phosphate-solubilizing bacteria strain with high Pb and Cr tolerance was isolated from a heavy metal contaminated site in the suburbs of Hebei Province, China. Phosphate-solubilizing microorganisms were screened by a plate assay method using Pikovskaya agar medium [24]. The lowest concentration of heavy metals that cause total inhibition of growth is known as minimum inhibitory concentration (MIC) [6]. The MIC for Pb and Cr of each microorganism were determined used for selecting the most resistant strains. Pb(NO_3_)_2_ and K_2_Cr_2_O_7_ were added separately to Pikovskaya agar medium with various concentration (0, 0.1, 0.5, 1, 2, 4, 8, 12, 24, 48, 72 mmol·L^−1^). The plates were incubated at 28 °C for 7 days and visually inspected for microbial growth. The isolated PSB strain was identified as *Bacillus* species by 16S rRNA sequencing (performed at Novogene Biotechnology Co., Ltd. Beijing, China). The strain was grown in Luria-Bertani (LB) agar plates and then incubated and stored on slants at 4 °C for subsequent experiments and preserved at −80 °C freezer for further study. 

### 2.2. Bacterial Growth

The isolated strain was cultivated in advance to reach logarithmic phase. And then triplicate 150 mL Erlenmeyer flasks containing 50 mL of autoclaved LB medium were inoculated with 1 mL (10^7^ CFU·mL^−1^) of inoculum and supplemented with 0.5, 1, 5 mmol·L^−1^ of Pb or Cr (Pb(NO_3_)_2_, K_2_Cr_2_O_7_), respectively. Cultures were then incubated at 37 °C under 120 rpm for 24 h to carry out batch experiments. Cultures grown in the absence of metal were used as control. 

Bacterial growth was measured as optical density at 600 nm (OD_600_) by a UV-Vis spectrophotometer (DR3900, Hach, Loveland, CO, USA). In order to discard the OD value due to Pb precipitates that formed by LB media components, using the LB media that added with corresponding concentrations of Pb as blank [25]. 

### 2.3. Quantification of ROS

The generation of intracellular reactive oxygen species (ROS) was determined using 2.7-dichorofluorescin diacetate (DCFH-DA) [26]. DCFH-DA passively enters the cell and reacts with ROS, to form the highly fluorescent compound DCF. After 24 h exposure, 10 mL of the culture were centrifuged at 3200 g for 10 min at 4 °C, the cell samples were collected and then rinsed with fresh phosphate buffer saline (50 mmol·L^−1^, pH 6.8), resuspended in phosphate buffer saline containing 4 µM DCFH-DA and incubated in darkness for 30 min at 37 °C. Cells were then washed out by phosphate buffer saline twice and transferred to 5 mL polystyrene round-bottom tubes (Falcon, BD, USA) for analysis of intracellular fluorescence by flow cytometry (FACSCalibur, BD, USA).

### 2.4. Superoxide Dismutase (SOD) & Catalase (CAT) Activity Analyses

After 24 h exposure, samples were centrifuged at 3200 g for 10min at 4 °C and the pellets were suspended in 3 mL phosphate buffer saline (50 mmol·L^−1^, pH 6.8). Suspended cells were then disrupted by submitting them to a vortex for 5 min, in the presence of 1 g of glass beads (0.1 mm diameter). The disruption period was 1min by 30 s interval in an ice bath. Cell debris and glass beads were then removed by centrifugation for 10 min (3200 g, 4 °C) and the supernatant was used for determinations of enzyme activity. 

SOD and CAT were determined using commercial kits (Nanjing Jiancheng Bioengineering Institute, Nanjing, China) and procedures were performed according to the manufacturer’s instructions (hydroxylamine method for SOD and ammonium molybdate method for CAT). Activities of SOD and CAT were expressed as units per milligrams of protein (U·mg^−1^ protein). Determined at 550 nm and 405 nm using UV-Vis spectrophotometer (DR3900, Hach, Loveland, CO, USA), respectively. 

### 2.5. Heavy Metal Bioaccumulation Assays

The heavy metal accumulation inside and outside the cell were determined according to Li [27] with some modifications. Briefly, after exposure for 24 h, 10 mL of the culture were centrifuged at 3200 g for 10 min and the bacterial cell in the pellet washed twice with ultrapure water, resuspended for 30 min in 10 mL 20 mmol·L^−1^ Ethylenediamine tetraacetic acid disodium magnesium salt (EDTA-2Na) for desorption of metal ions from the cell surface. Then centrifuged to remove supernatant and had cells weighed after drying to constant weight at 80 °C for 24 h. The amount of heavy metals bioaccumulated intracellularly and extracellularly was determined by the digested bacterial cells and the supernatant, respectively. The metals were quantified by an inductively coupled plasma mass spectrometry (ICP-MS) (7900, Agilent, Santa Clara, CA, USA).

### 2.6. Scanning Electron microscopy (SEM) and Scanning Transmission Electron Microscopy-Energy Dispersive X-ray Spectroscopy (STEM-EDS)

Samples were obtained by centrifugation at 3200 g (4 °C) for 10min after 24 h exposure to get pellets and washed twice with phosphate buffer saline (50 mmol·L^−1^, pH 6.8), subsequently fixed in 2.5% glutaraldehyde for 18 h at 4 °C. After fixation, pellets were washed again using deionized sterile water, serially dehydrated with ethanol. For SEM, pellets were further dehydrated by using a lyophilizer. A fraction of lyophilized cells was then placed on a brass stub, coated with gold, examined by SEM (SU8010, Hitachi, Japan). For STEM, pellet was further dehydrated using acetone and embedded in Suprr’s resin. Ultrathin sections of 90–110 nm thickness were cut using an ultramicrotome and taken on a copper grids in a transmission electron microscopy (JEM-2100F, JEOL, Japan) operated at 200 kV. EDS was employed with the same instrument.

### 2.7. Attenuated Total Reflectance Fourier Transform Infrared Spectroscopy (ATR-FTIR) and X-ray Photoelectron Spectrometer (XPS) Analyses

The chemical characteristics of bacterial cells were analyzed using an attenuated total reflectance Fourier transform infrared (ATR-FTIR) (Vertex, Bruker Optics, Germany) to confirm whether heavy metals had been entrapped within the functional groups. All infrared spectra were recorded over the range of 4000–400 cm^−1^. Furthermore, the investigation of chromium valence states that distributed outside the bacterial cell were carried out by using an Esca Lab 250Xi spectrometer (Thermo Fisher, USA) equipped with an Al anode (Al-Kα excitation) as X-ray source. Samples were obtained by centrifugation at 3200 g (4 °C) for 10min and got the supernatant removed. This low centrifugation speed has been utilized as a model procedure to separate intact bacteria. For loosely bound EPS, the pellets were then resuspended with 1 mL ultrapure water after the steps above and centrifuged at 12,000 g (4 °C) for 10 min, the supernatant was collected and dehydrated by lyophilizer to get the samples for ATR-FTIR and XPS. 

### 2.8. Statistical Analysis

All experiments were performed in triplicate and the data were expressed as mean ± standard deviation. The statistical analyses were performed with one-way analysis of variance (ANOVA) followed by Tukey’s multiple comparison and statistical significance was selected as *p* < 0.05.

## 3. Results & Discussion

### 3.1. Effect of Pb and Cr Stress on PSB Growth

**Figure 1:**
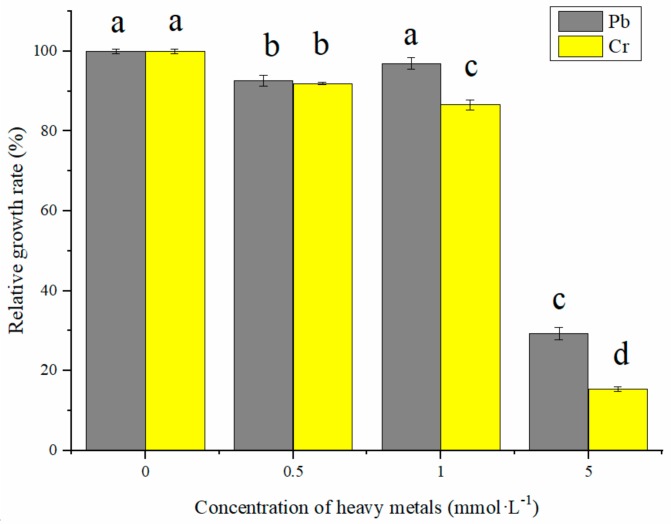
Relative growth rate of *Bacillus* sp. MRP-3 under Pb and Cr stress compared with control group. Bars with different letters (a–d) are significantly different at *p* < 0.05.

The MICs of Pb and Cr were in 8 mmol·L^−1^ and 4 mmol·L^−1^, respectively. To further investigate the effects of heavy metals on bacterial growth, different concentrations of Pb or Cr were added to the LB media and the growth of *Bacillus* sp. MRP-3 was measured after 24 h exposure. The relative growth rate (% of control) was used to analyze the effect of different metal stress conditions on the growth of the bacterial cells. As Figure 1 shows, there was no evidently suppression up to 1 mmol·L^−1^ concentration of Pb or Cr, which suggests that isolated strain MRP-3 has strong ability to tolerate heavy metals. However, bacterial growth significantly declined under 5 mmol·L^−1^ concentrations of Pb and Cr, with the relative growth rate decreased to 29.2% and 15.3% respectively, indicating that high concentrations of Pb and Cr were toxic for bacterial cells. The strain growth rate was lower in Cr exposure than Pb at the same concentration, which implies that the isolated strain was more sensitive to Cr than Pb. The decrease of the relative growth rate can be attributed to the bioavailability and toxicity of the metals. *Bacillus* sp. was widely reported as potential bioremediation bacteria for its heavy metal tolerance. Shin [28] reported *Bacillus* sp. MN 3-4 was excellent in Pb tolerance and accumulation at 100 mg·L^−1^ concentration; Mahmood [29] reported that *Bacillus* sp. SR-2-1/1 tolerated high concentrations of Cr up to 1000 mg·L^−1^ and acted as plant growth promoting rhizobacteria. Besides, *Bacillus* sp. had been reported as proper PSB to promote plant growth and enhance phytoremediation [5]. 

**Figure 2:**
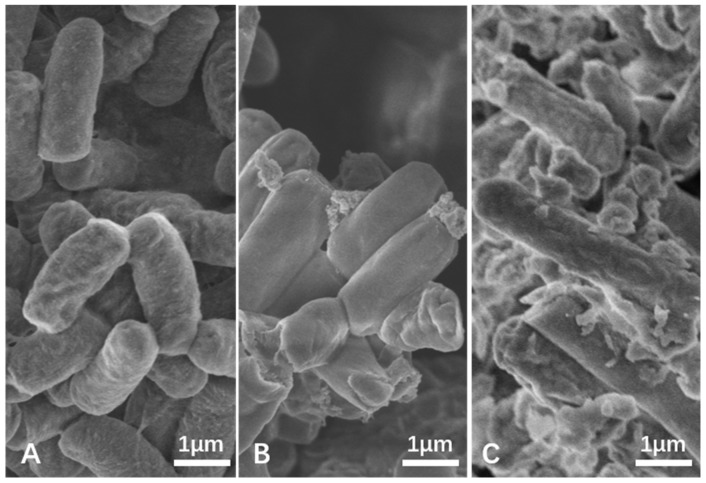
SEM images of *Bacillus* sp. MRP-3 under Pb and Cr stress ((**A**): control group, (**B**): 1 mmol·L^−1^ Pb, (**C**): 1 mmol·L^−1^ Cr).

The morphological differences of *Bacillus* sp. MPR-3 in the presence and absence of the Pb and Cr were examined by SEM (Figure 2). It shows that bacteria grew with aggregate formation and intact morphological features in the medium without heavy metals (Figure 2a). Whereas, cells under heavy metals show aggregate formation of cells, disruption of cell wall, and highly distorted morphological features (Figure 2b,c). As compared to the control, Figure 2b shows that cells became bloated, smooth, and formed precipitates in the presence of Pb. Figure 2c shows that cells became twisted with stretched cell size and surrounded by numerous cell debris due to the addition of Cr. The distinct morphological alterations, deformation, and severe membrane damage, all these results indicate Pb and Cr toxicity to bacteria that result in different degrees of damage. Additionally, cell debris could be the secretion of a matrix of exopolysaccharide in order to protect cell from metals or in order to retain metals by adsorption and prevent accumulation into the cells [30,31]. 

### 3.2. Effect of Pb and Cr on Oxidative Stress

**Figure 3:**
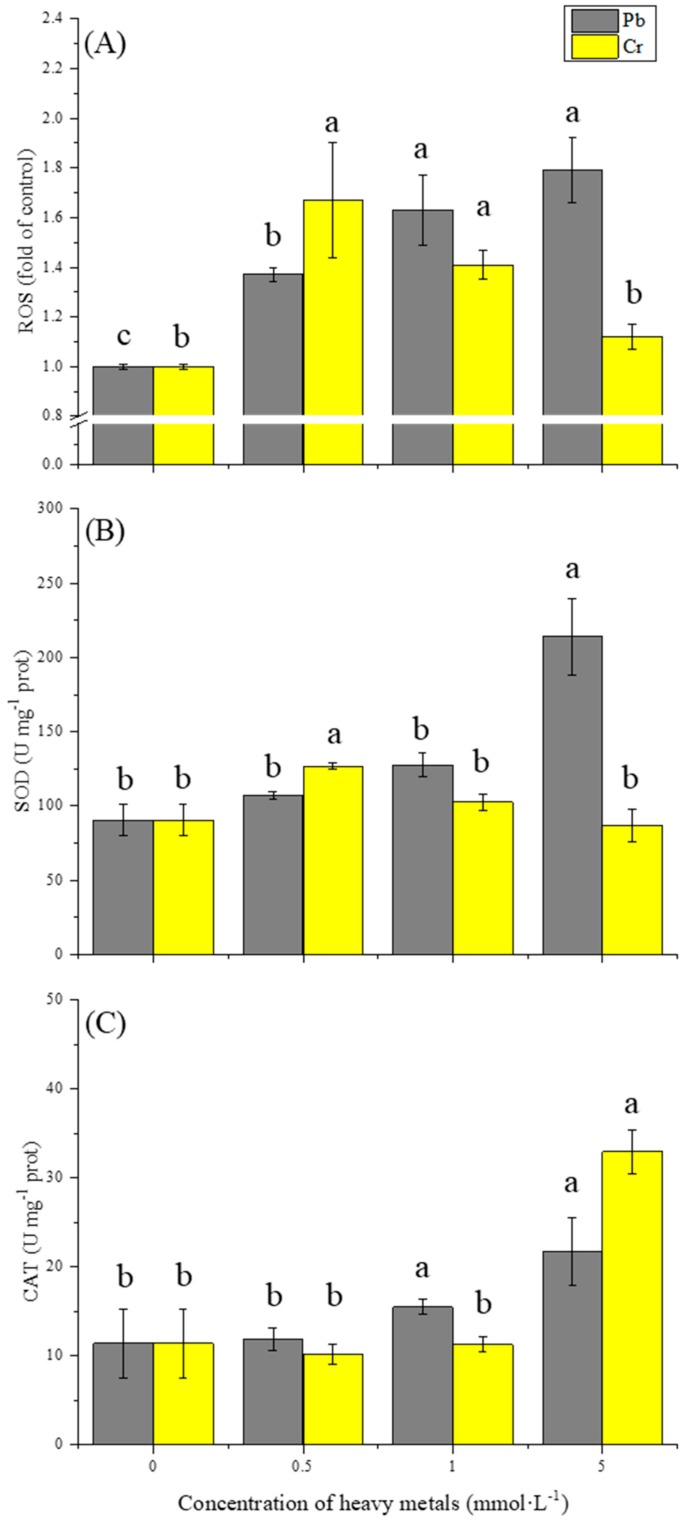
Effects of Pb and Cr on ROS generation (**A**), antioxidative enzymes activities of SOD (**B**) and CAT (**C**). Bars with different letters (a–c) are significantly different at *p* < 0.05.

For revealing more facts about different relative growth rate under corresponding metal conditions, intracellular oxidative stress was investigated (Figure 3). Different concentrations of Pb or Cr were added to the media and intracellular ROS was measured after 24 h exposure. Pb promoted a marked increase in ROS levels in a gradual concentration-dependent manner, with 1.4-fold of control in 0.5 mmol·L^−1^ condition, 1.6-fold of control in 1.0 mmol·L^−1^ condition and 1.8-fold of control in 5.0 mmol·L^−1^ condition (Figure 3A). It revealed intracellular ROS could be induced by Pb and has a concentration-mannered behavior. In contrast, the lowest concentration of Cr (0.5 mmol·L^−1^) triggered a significant rise in the formation of intracellular ROS, and this signal was quickly declined after exposure to higher concentrations of Cr. For the highest ROS level under Cr condition (0.5 mmol·L^−1^), only a slight bacterial growth inhibition existed. However, for the lowest ROS level under Cr condition (5 mmol·L^−1^), the bacterial growth was suppressed obviously. The ROS generation during 24 h exposure under Cr stress does not associate with the considerable toxicity to *Bacillus* sp. MRP-3 as observed in bacteria growth (Figure 1) which indicated that ROS may not be the main factor to effect cell viability under Cr stress. 

The phenomenon that heavy metals induce ROS and result in growth inhibition has been observed before. Kováčik [17] reported that Pb posed the rapid increase of ROS in *Coccomyxa subellipsoidea* which inhibited cell viability. Li [15] reported that *Pleurotus ostreatus* HAU-2 growth was suppressed by high concentrations of Cr, because of induced ROS. However, in this study, where it was suggested that Pb and Cr could affect microorganisms and cause different effect in terms of ROS, i.e., under higher Cr stress, fewer oxidative stress but more distinct growth inhibition was measured. And similar reports were found by Zhang [32] that Zn(II) led to a lower ROS level under high concentrations in *E.coli* K12. This might be attributed to the fact that the cytotoxicity of Cr(VI) cannot be solely explained by the action of ROS; intracellular biological reductants could reduce Cr(VI) to Cr(III) inside the cell, the latter interferes with DNA and affects replication [9].

This result indicates that both Pb and Cr could contribute to the increase of ROS, causing excess oxidative stress inside the cell. At lower concentrations, Cr(VI) caused excessive ROS generation when compared to Pb(II) but without obvious inhibition of bacterial growth, which implies that Cr ions could affect intracellular oxidative balance via tiny doses than Pb. Higher Pb ion concentration could continuously induce the increasing of ROS, which results in higher toxicity. The decline of ROS level under higher Cr stress maybe attributed to the damage of oxidative balance. Our study confirmed that the growth inhibition cannot be solely explained by the action of ROS. 

### 3.3. Effect of Pb and Cr on Antioxidant Enzymes

SOD and CAT were measured to investigate the antioxidant enzymes activities during the excess oxidative stress. As shown in the Figure 3b and c, the isolated bacterium possessed basal levels of SOD and CAT activity in the absence of heavy metals. The SOD activity of strain was increased by both Pb and Cr in the first place, but further increased when a larger amount of Pb was added in the medium. The maximal SOD activity was observed at concentration of 5 mmol·L^−1^ Pb (210 U·mg^−1^ protein), in which the SOD activity was 2.1-fold higher than that of the control group (89 U·mg^−1^ protein), presented a Pb-concentration-dependent manner (Figure 3B). Whilst SOD activity in Cr stress was found to decrease according to concentration increasing. For Cr stress, SOD activity was found to increase first but decreased with higher concentration. The highest SOD level was recorded at 0.5 mmol·L^−1^ (125 U·mg^−1^ protein), declined to 100 U·mg^−1^ protein at 1 mmol·L^−1^ and 90 U·mg^−1^ protein at 5 mmol·L^−1^. In addition, the variation of SOD activity under both metals shown a ROS-dependent manner, which indicated SOD played a significant role in removing ROS. SOD activity was found negatively correlated with Cr concentration (Figure 3B), and similar observation was also reported [15,33]. Li [15] attributed this phenomenon to the underlying suppression of enzyme synthesis with overly high Cr stress. However, our study implied that it was more likely that the induced ROS decreased in the first place, which might have contributed to Cr toxicity, and SOD decreased consequently. 

In contrast to SOD, increasing trends were observed in CAT activity under both Pb and Cr stress (Figure 3C). Pb and Cr did not contribute to the increase of CAT activity at lower concentrations. The CAT activity reached to 15.5 and 11.2 U·mg^−1^ protein at Pb and Cr concentration of 1 mmol·L^−1^, but then the CAT activity rose obviously to 21.8 and 32.9 U·mg^−1^ protein at Pb and Cr concentration of 5 mmol·L^−1^, respectively. The results shown CAT presented a concentration-dependent manner. Rai [33] reported similar observation in *Chlorella* under Cr stress. CAT catalyzes the decomposition of H_2_O_2_ to H_2_O and O_2_, as a very important enzyme in protecting the cell from the oxidative damage by ROS. The phenomenon that CAT activity will not rise at slight stress but increases rapidly under severe conditions may indicated more H_2_O_2_ produced after metabolic process under a severe condition compared with slight stress. It suggests that CAT activity increases along with the increase of heavy metal stress, instead of the oxidative stress, especially in Cr stress. The experiment demonstrated that the production of antioxidant enzymes is a biochemical means by which organisms respond to metal accumulation and microorganisms may appear to have different sensitivity and response to different metals in terms of oxidative stress.

### 3.4. Effect of Pb and Cr on Bioaccumulation

Intracellular accumulation and extracellular absorption were shown in Table 1. For extracellular adsorption, the amount of heavy metal ions was increased with concentrations under both Pb and Cr stress. An obvious accumulation occurred on the cell surface for Pb ions, absorption capacity could be up to 0.211 mmol·g^−1^ just at lowest concentration (0.5 mmol·L^−1^) and get to 1.318 mmol·g^−1^ at highest concentration (5 mmol·L^−1^). In contrast, there were little ions absorbed for Cr ions, only 0.021 mmol·g^−1^ could be measured even at highest concentration (5 mmol·L^−1^). It indicated that the substances outside the cell could absorb or react with Pb ions, whilst the capacity to absorb Cr ions is very limited. One of the mechanisms for microbes to exclude the toxic metals is to limit their movement across the cell envelope [21]. Metal ions can be absorbed through electrostatic attraction and ion-exchange by the functional groups on the cell wall or extracellular polymeric substances, which can prevent vital cellular components from interacting with metal ions. Pb has been detected to have strong affinity with extracellular polymeric substances, which relate to hydration energy and complexation [34], while Cr may have limited affinity with surface functional groups. 

For intracellular accumulation, higher concentrations of metal ions was observed at higher Pb and Cr stress, up to 0.558 and 0.565 mmol·g^−1^, respectively, implying that cell’s intracellular accumulation were affected significantly by the surrounding metal concentrations. Many previous studies had reported that heavy metal toxicity was usually related to the intracellular heavy metal concentration, and the heavy metal tolerance was improved or reduced by weakening or enhancing heavy metal bioaccumulation [27]. The bacterial cells cultivated in Pb had less intracellular accumulation than Cr at any equal concentration, it might be accounted for that lots of Pb ions had been adsorbed outside the cell, which may also be an important reason for the higher bacterial growth rate of *Bacillus* sp. MRP-3 under Pb stress (Figure 1). 

Interestingly, these results provided an unusual conclusion against other reports that the production of ROS was thought to be positively correlated with heavy metal accumulation in organisms [35]. In this study, with the rising of Cr stress level, it was found that the intracellular accumulation increased but ROS decreased, with SOD activity decreased too. The results indicated that oxidative stress could be induced by relatively low concentrations of Cr, which caused antioxidant enzymes responses. However, overly high concentrations of Cr might damage cell metabolisms and consequently interfere with oxidative balance, accompanied by the decreasing of ROS. 

**Figure 4:**
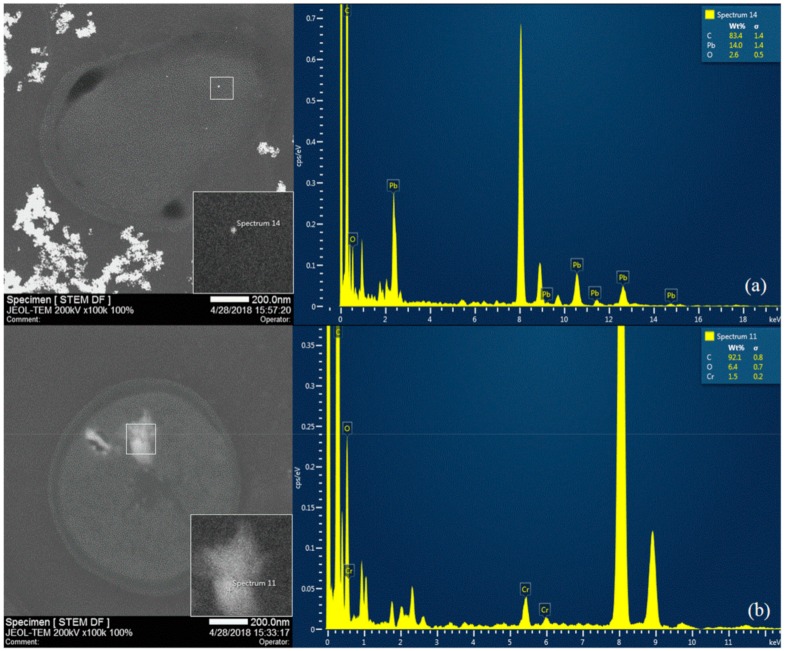
STEM-EDS of intracellular bioaccumulation images under 1 mmol·L^−1^ Pb (**a**) and Cr (**b**) stress.

To further investigate the intracellular accumulation and extracellular absorption of heavy metals, a STEM-EDS analysis was conducted. In Figure 4, there is an obvious closed region with a double-layer border in each STEM picture, which represents cell wall in the cross-section of cell. The bright spots situated in the internal region (labeled with ‘Spectrum 14’ and ‘Spectrum 11’) were proved to be Pb and Cr element by EDS. Figure 4 demonstrated that both Pb and Cr could be bioaccumulated inside the cell. Besides, it showed Pb precipitation could easily occur outside the cell, yet there was no precipitation under Cr condition.

### 3.5. ATR-FTIR Spectroscopy and XPS Analyses 

From the results of adsorption in Table 1, the extracellular Pb was 27 times higher than Cr under 1 mmol·L^−1^ concentration, and 63 times higher than Cr under 5 mmol·L^−1^ concentration. Among the extracellular adsorption, EPS was thought to be a key role in adsorption, amidst this, loosely bound EPS was reported to have more important role in bacterial properties [22,23]. To test the hypothesis that loosely bound EPS had been involved in heavy metal adsorption, ATR-FTIR was conducted to investigate intact bacteria and loosely bound EPS. 

**Figure 5:**
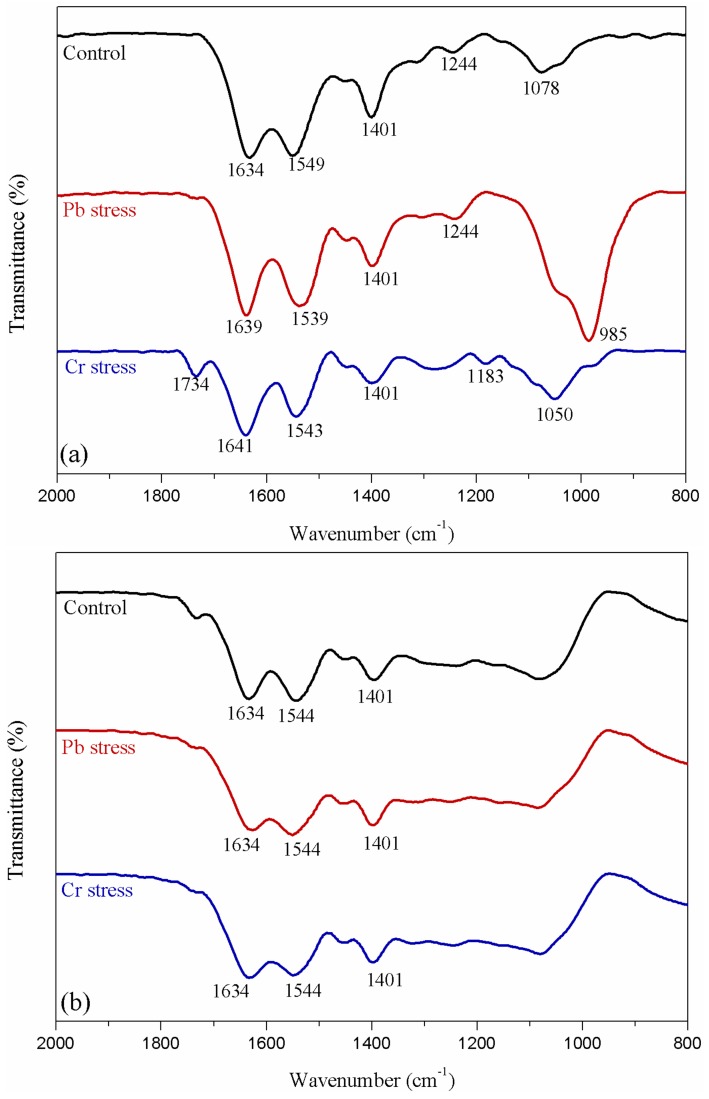
ATR-FTIR spectra of intact bacteria (**a**) and loosely bound EPS (**b**) under 1 mmol·L^−1^ Pb and Cr stress.

Figure 5 shows infrared spectra of bacterium under both heavy metal conditions from 800 to 2000 cm^−1^. The peak assignments were as follows [23,34,36,37]: ~1634 cm^−1^ (C=O and C–N stretching in Amide I); ~1549 cm^−1^ (C–N stretching vibration and N–H deformation vibration of Amide II); ~1401 cm^−1^ (C=O symmetric stretching of carboxylic groups); ~1244 cm^−1^ (PO_2_^−^ stretching from phosphodiesters and phosphate groups); ~1078 cm^−1^ (P–O stretching from phosphoryls). For intact bacteria (Figure 5a), bands at 1634 cm^−1^ shifted to higher wavenumber and bands at 1549 cm^−1^ shifted to lower wavenumber, indicating C=O, C–N and N–H group bonded heavy metals in the adsorption process. By comparison, ATR-FTIR for loosely bound EPS (Figure 5b) presented three main peaks at 1634 cm^−1^, 1544 cm^−1^, and 1401 cm^−1^, but without visible peak shift. This result demonstrated that different functional groups were involved in Pb or Cr adsorption while these functional groups might not locate in loosely bound EPS. Notably, the bands at 1078–1244 cm^−1^ range did not present in Figure 5b, implying that these characteristic peaks might belong to the rest parts of the bacteria.

**Figure 6:**
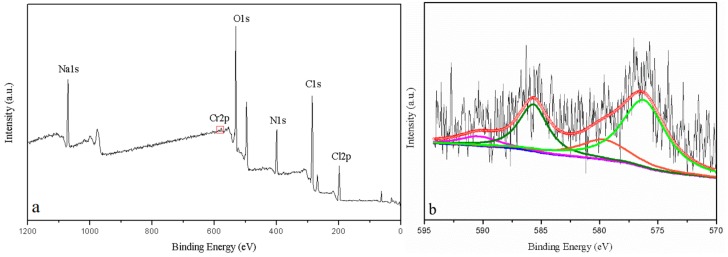
The survey (**a**) and high resolution (**b**) XPS spectra of Cr 2p in EPS. The peaks at 585.7 eV and 576.3 eV can be assigned to Cr 2p_1/2_ and Cr 2p_3/2_ for Cr(III) respectively, the peaks at 590.4 eV and 579.8 eV can be assigned to Cr 2p_1/2_ and Cr 2p_3/2_ for Cr(VI) respectively.

The reduction of Cr(VI) is known as a key detoxification mechanism, i.e., direct reduction via electron-donor group in the aqueous phase; indirect reduction via binding of Cr(VI) anionic species to positively charged functional groups on the biomass surface, and then Cr(VI) get reduced to Cr(III). Investigation of valence state of chromium outside bacterial cell would be useful to demonstrate the interaction between *Bacillus* sp. MRP-3 and heavy metals. XPS survey of loosely bound EPS under Cr stress (growth in 1 mmol·L^−1^ concentration) was conducted and analyzed according to previous studies [38]. The peaks of Cr could not be identified in survey spectrum which might be attributed to the low contents of metal ions in the extracellular substance (Figure 6a,b). The high resolution of Cr 2p spectrum comprised with four peaks corresponding to binding energy of Cr 2p_1/2_ and Cr 2p_3/2_ states. Peaks at 585.7 eV and 576.3 eV can be assigned to Cr 2p_1/2_ and Cr 2p_3/2_ for Cr(III) respectively, indicating the formation of Cr(III). The presence of Cr(III) may due to the extracellular reduction by the living cells or release from the death cells. Peaks at 590.4 eV and 579.8 eV can be assigned to Cr 2p_1/2_ and Cr 2p_3/2_ for Cr(VI), respectively, suggesting that there remains Cr(VI) outside the bacterial cells (Figure 6b). Previous studies found that Cr(VI) adsorption increased at low pH when functional groups on the cell surface became protonated and easily attract negatively charged Cr species (CrO_4_^2−^/Cr_2_O_7_^2−^) [9,39]. In this study positive charged functional groups were limited under the culture condition (pH ~7), which might have led to low Cr adsorption. 

## 4. Conclusions

The growth suppression cannot be solely explained by the action of ROS—the intracellular ROS under Cr(VI) stress was found negatively correlated with growth suppression and intracellular accumulation. SOD activities were induced following the ROS variation under both Pb(II) and Cr(VI) stress, which implied that SOD played a significant role in removing of ROS, yet CAT activity tended to increase with heavy metal stress instead of ROS variation. The extracellularly accumulated Pb accounted for 70.3–95.9% of the total accumulation, while extracellularly accumulated Cr only accounted for 1.3–3.6% of the total accumulation. The disparity in extracellular accumulation may be the result of Pb possessing lower cytotoxicity than Cr at same concentration. Functional groups played an important role during the process of extracellular adsorption, but the functional groups that are located in loosely bound EPS was not involved in the adsorption. Pb(II) and Cr(VI) have different effects on strain’s oxidative stress and intra/extracellular accumulation behavior, and the bacterial tolerance to heavy metals was related to the ability to reduce oxidative stress and metal accumulation inside the cell. Further study will focus on the relationship between heavy metal stress and capability of phosphorus mobilization of the phosphorus solubilizing bacteria strain.

## Figures and Tables

**Table 1:** Heavy metal intracellular and extracellular bioaccumulation (mmol·g^−1^ dry weight) of *Bacillus* sp. MRP-3 under different Pb and Cr stress. Values in the same column with different letters (a–c) are significantly different at *p* < 0.05.

Initial Metal Concentration (mmol·L^−1^)	Pb Accumulated (mmol·g^−1^ dry weight)		Cr Accumulated (mmol·g^−1^ dry weight)	
	Intracellular	Extracellular	Intracellular	Extracellular
0.5	0.009 ± 0.002 ^c^ (4.1%)	0.211 ± 0.031 ^b^ (95.9%)	0.034 ± 0.002 ^c^ (98.7%)	<0.000 ^b^ (1.3%)
1.0	0.134 ± 0.008 ^b^ (38.3%)	0.216±0.005 ^b^ (61.7%)	0.258 ± 0.026 ^b^ (97.0%)	0.008 ± 0.002 ^b^ (3.0%)
5.0	0.558 ± 0.035 ^a^ (29.7%)	1.318±0.285 ^a^ (70.3%)	0.565 ± 0.036 ^a^ (96.4%)	0.021 ± 0.008 ^a^ (3.6%)

Values in parentheses indicate the percent of total heavy metal bioaccumulation.

## Abbreviations

**Abbreviations Used in the Text:** phosphate-solubilizing bacteria (PSB), reactive oxygen species (ROS), superoxide dismutase (SOD), catalase (CAT), extracellular polysaccharides substances (EPS), inductively coupled plasma mass spectrometry (ICP-MS), scanning electron microscopy (SEM), scanning transmission electron microscopy -energy dispersive X-ray spectroscopy (STEM-EDS), attenuated total reflectance-Fourier transform infrared (ATR-FTIR), X-ray photoelectron spectrometer (XPS).
